# Whole body vibration for chronic patellar tendinopathy: A randomized equivalence trial

**DOI:** 10.3389/fphys.2022.1017931

**Published:** 2022-10-21

**Authors:** Florian Rieder, Hans-Peter Wiesinger, Jürgen Herfert, Katrin Lampl, Stefan Hecht, Josef Niebauer, Nicola Maffulli, Alexander Kösters, Erich Müller, Olivier R. Seynnes

**Affiliations:** ^1^ Institute of Physical Medicine and Rehabilitation, Paracelsus Medical University Salzburg, Salzburg, Austria; ^2^ Department of Sport and Exercise Science, Paris-Lodron University Salzburg, Salzburg, Austria; ^3^ Red Bull Athlete Performance Center, Thalgau, Austria; ^4^ Institute of Radiology, Paracelsus Medical University Salzburg, Salzburg, Austria; ^5^ Institute of Sports Medicine, Prevention and Rehabilitation, Paracelsus Medical University Salzburg, Salzburg, Austria; ^6^ Department of Musculoskeletal Disorders, Faculty of Medicine and Surgery, University of Salerno, Salerno, Italy; ^7^ Centre for Sports and Exercise Medicine, Barts and the London School of Medicine and Dentistry, Mile End Hospital, Queen Mary University of London, London, United Kingdom; ^8^ School of Pharmacy and Bioengineering, Keele University School of Medicine, Staffordshire, United Kingdom; ^9^ Department of Physical Performance, Norwegian School of Sport Science, Oslo, Norway

**Keywords:** VISA-P, VAS, tendon mechanical properties, tendon morphology, jumpers knee, heavy slow resistance training, MRI

## Abstract

**Purpose:** Whole body vibration (WBV) triggers anabolic responses in various tissues, including tendons, without requiring high force production. In this waitlist-controlled equivalence trial, we tested its clinical effectiveness as an alternative treatment for patellar tendinopathy against conventional heavy slow resistance training (HSR).

**Methods:** Thirty-nine patients were randomized to either 3 months of WBV training (*n* = 13), HSR training (*n* = 11), or a waitlist control (WLC) group (*n* = 15). In a partly cross-over design, 14 patients of the WLC group were redistributed to one of the two intervention groups (5 in WBV, 9 in HSR). Pre- and post-intervention testing included pain assessments (VAS), functional limitations (VISA-P), knee extension strength and tendon morphological, mechanical and material properties. Follow-up measurements (VAS, VISA-P) were performed in the WBV and HSR groups 6 months after the intervention.

**Results:** Comparisons with the WLC group revealed significant improvements in VISA-P and VAS scores after HSR (41%, *p* = 003; 54%, *p* = 0.005) and WBV (22%, *p* = 0.022; 56%, *p* = 0.031) training. These improvements continued until follow-up (HSR: 43%, 56%; WBV: 24%, 37%). Pre-post improvements in VAS scores were equivalent between WBV and HSR groups but inconclusive for the VISA-P score and all pre-test to follow up comparisons. The mid-tendon cross-sectional area was significantly reduced after WBV (−5.7%, *p* = 0.004) and HSR (−3.0%, *p* = 0.004) training compared to WLC although the equivalence test between interventions was inconclusive.

**Conclusion:** Whole body vibration improved symptoms typically associated with patellar tendinopathy. This type of intervention is as effective as HSR against maximum pain, although equivalence could not be confirmed for other variables. The beneficial responses to WBV and HSR treatments persisted for 6 months after the end of the intervention.

**Clinical Trial Registration:** https://www.drks.de/drks_web/setLocale_EN.do, identifier DRKS00011338

## Introduction

Patellar tendinopathy is common in athletes, leading to persistent pain, functional limitations or even ending of sporting career (Maffulli et al., 2003). It is often attributed to an overuse syndrome (Cook and Purdam, 2009), although its aetiology is largely unknown. Histologically, the pathology is a failed healing response of the extracellular matrix, with thinner, disorganized collagen fibres (Kongsgaard et al., 2010), tendon swelling and often neovascularisation (Öhberg et al., 2001). In some cases, histological and morphological changes accompany a decrease in stiffness of the Achilles (Child et al., 2010) or patellar tendon (Wiesinger et al., 2020). Exercise-based treatments, shockwave therapy, or platelet-rich plasma injections are currently the most common non-surgical approaches to treat patellar tendinopathy (Andriolo et al., 2018). Eccentric-only or eccentric-concentric loading regimes are the conservative strategies of choice, being non-invasive and cost-effective (Larsson et al., 2012). Some training-based protocols were shown to reverse histopathological features associated with tendinopathy (Kongsgaard et al., 2009; Kongsgaard et al., 2010).

Nonetheless these interventions are very time-consuming with two training sessions twice a day for the eccentric (duration of about 10 min for each session) and three sessions per week with 45–60 min duration for the eccentric-concentric protocols (Kongsgaard et al., 2009). For elite athletes, rehabilitation must be conducted concurrently with training and competition, and such protocols may not be well accepted or might even compromise the effectiveness of treatment (Visnes et al., 2006; van Ark et al., 2015). Additionally, the prescribed slow exercises (3 s for the eccentric and 3 s for the concentric part (Kongsgaard et al., 2009)) often conflict with the contractile profile found in explosive sports, and may therefore lead to decreases in the production of mechanical power (Stasinaki et al., 2019).

Whole body vibration (WBV) is based on sinusoidal oscillations from a source platform to a standing individual. One training session usually lasts about 19 min, while the active part lasts 10 min and is performed 2–3 times per week (Cochrane 2011). Vibration stimuli, transmitted within safe frequency and amplitude ranges, induce remodeling and anabolic responses in muscles (Gilsanz et al., 2006) and bones (Verschueren et al., 2004; Gilsanz et al., 2006) of the lower limb. The anabolic effects of vibration exposure were also observed in animal studies on tendinous tissue, resulting in enhanced collagen synthesis (Thompson et al., 2015) and increased tendon cross-sectional area (CSA) and stiffness (Sandhu et al., 2011). Our group recently reported regionally increased patellar tendon CSA after WBV training in healthy adult men and women (Rieder et al., 2016), similarly to the hypertrophic responses reported following heavy resistance training in a comparable cohort (Kongsgaard et al., 2007; Seynnes et al., 2009). Therefore, WBV training likely induces similar remodeling and healing effects as ascribed to the strains imposed by heavy loads (Kongsgaard et al., 2010). In support of this hypothesis, reductions in Achilles tendon pain following vibration training, coupled with progressive concentric-eccentric heel raises, were recently reported (Horstmann et al., 2013). Despite these promising results, the clinical effectiveness of WBV as a sole intervention remains unexplored.

We investigated the effects of WBV on tendon pain and functional limitations, on ultrasonographic features associated with tendinopathy (vascularization, tendon thickening and hypoechoic areas), knee extension strength and mechanical and material properties in patients with patellar tendinopathy, and compared WBV to a heavy slow resistance loading program (HSR (Kongsgaard et al., 2009)) and a waitlist control group (WLC). We hypothesized that both interventions would affect outcome variables, compared to the WLC group, but that HSR and WBV would be equally effective to attenuate symptoms of patellar tendinopathy. A further aim was to explore, if the expected clinical improvements are related to changes in tendon properties.

## Material and methods

### Study design

A randomized, single-blinded, waitlist controlled, equivalence trial was conducted at two sites between 2017 and 2019: the Institute of Physical Medicine and Rehabilitation of the Paracelsus Medical University Salzburg and the Department of Sport and Exercise Science of the Paris-Lodron University of Salzburg. Patients were recruited *via* local newspaper advertisements, social media, calls, and visits to local sports clubs. The study was registered at the German register for clinical trials (DRKS00011338) and was approved by the Local Ethics Committee Salzburg for medical research (415-E/2012/11-2016). It complied with the Declaration of Helsinki, and all subjects gave their written informed consent to participate. There was no change in the study design after commencement. The reporting of this equivalence trial follows the CONSORT guidelines (Piaggio et al., 2006) (see CONSORT checklist in Supplementary Table S1).

### Power analysis

The sample size for testing equivalence was calculated for the main clinical outcome variables (changes in VISA-P and visual analogue pain scale (VAS) scores) using a two-sided alpha level of 5% and a power of 80% (Julious, 2004). The equivalence margins (eΔ) were set at ± 13 for VISA-P scores (Hernandez-Sanchez et al., 2014) and ±1.8 for the VAS scores (Todd and Funk, 1996), with an expected standard deviation of ±11 and ±1.5, respectively (mean baseline value taken from the literature (Kongsgaard et al., 2009; Kongsgaard et al., 2010)). The estimated sample size was *n* = 13 per group for the VISA-P and *n* = 12 for the VAS score.

### Patients

**FIGURE 1:**
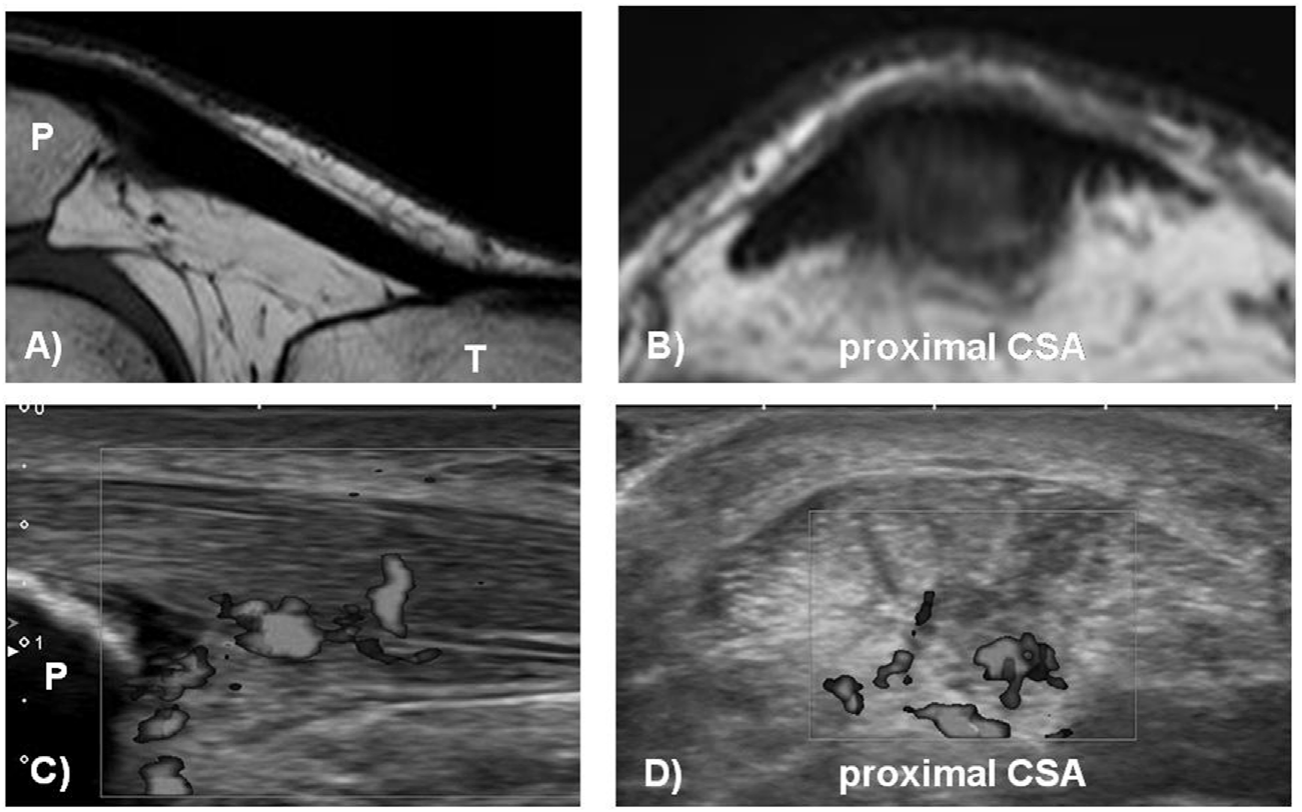
Magnetic resonance imaging [MRI, **(A,B)**] and ultrasound [US **(C,D)**] of tendinopathic patellar tendon in the sagittal and transversal plane showing tendon thickening [US **(C)** and MRI **(A)**], hypoechoic areas and neovascularisation **(C,D)** and increased signal intensity **(B)**. CSA = cross-sectional area; P = patella; T = tibia.

After a 4-week wash-out period of no treatment for chronic patellar tendinopathy (≥3 months), male and female patients between 18 and 40 years were randomized to WBV, HSR or WLC. The clinical diagnosis of patellar tendinopathy was conducted by experienced physicians (KL & JH). Screening for eligibility included patellar tendon pain (proximal and/or distal) in connection with loading during daily activities (e.g., ascending or descending stairs, jumping, landing, running), tenderness on palpation, and a VISA-P score lower than 87 (to allow for the minimum clinically meaningful improvement of 13 points (Hernandez-Sanchez et al., 2014)). In a second step, the initial screening was validated after the following ultrasonographic or magnetic resonance imaging (MRI) features associated with tendinopathy: antero-posterior tendon thickening (1 mm compared to the mid-tendon region) (Kongsgaard et al., 2009), hypoechoic areas (ultrasound, Logiq S7, General Electric, Solingen, Germany) and/or increased signal intensity (MRI, 3T-Achieva, Philips Healthcare, Eindhoven, Netherlands) (see Figure 1). The same experienced radiologist (SH) assessed all the scans collected in this step at the Paracelsus Medical University Salzburg. Exclusion criteria comprised cardiovascular diseases, orthopedic disorders of the affected leg, any injuries or degenerations of the knee joint not related to tendinopathy, inflammatory conditions of the musculoskeletal system, epilepsy, diabetes, pregnancy, tumors, any implant held in place by a magnet, eye lenses, claustrophobia, injections in the patellar tendon in the preceding 3 months, corticosteroid injections within 12 months, current use of anticoagulants. Concomitant knee joint injuries were excluded using comprehensive manual clinical examinations (e.g., pivot shift test, Steinmann II test, Lachman test). Patients with bilateral symptoms received the same treatment on both sides, and the most symptomatic knee (lowest VISA-P score) was selected for measurements.

### Randomization and blinding

**FIGURE 2:**
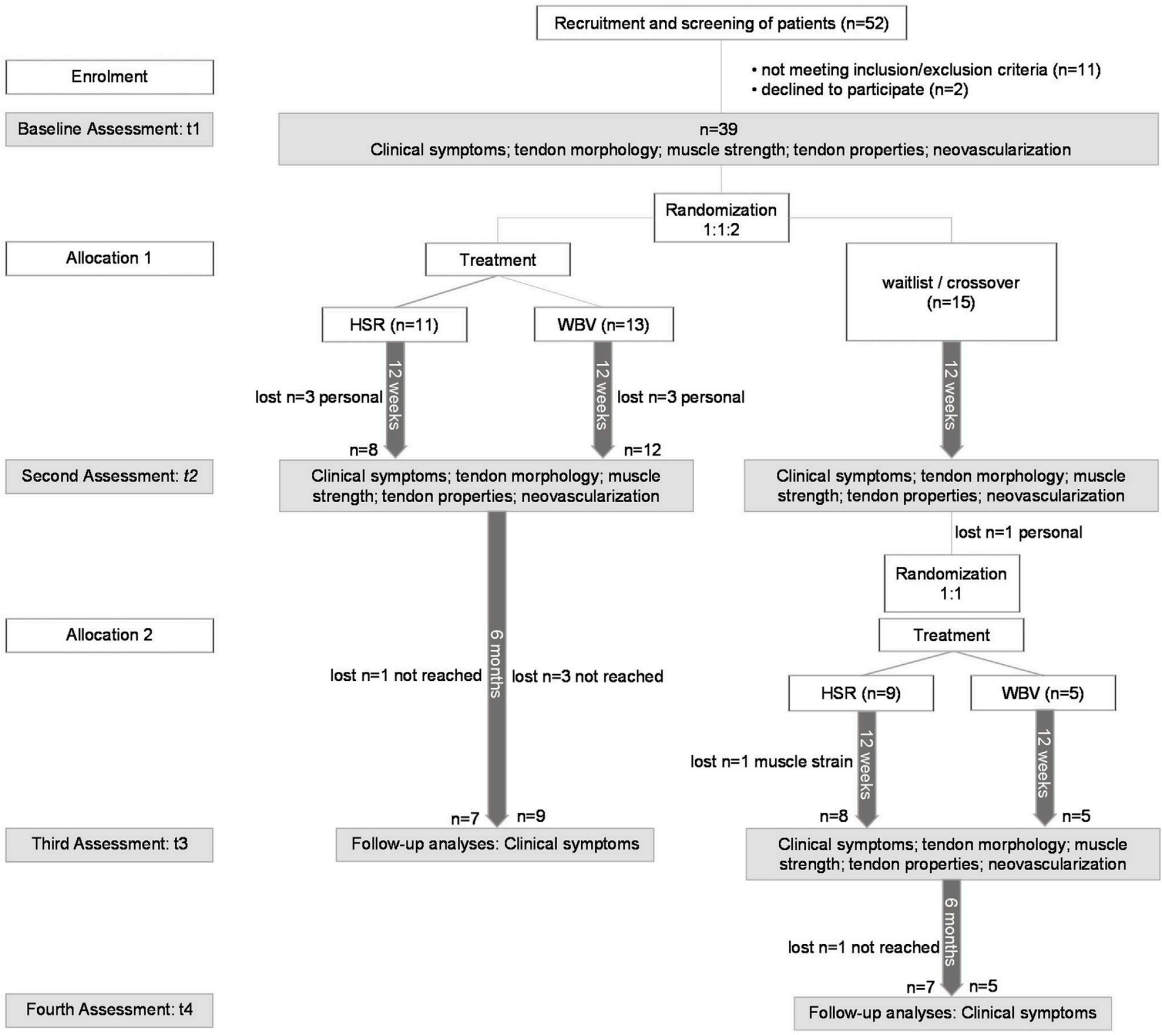
Study flow. HSR = heavy slow resistance training; WBV = whole body vibration training. Personal = drop out due to personal reasons: moved away/no time for the study; not reached = patient could not be reached by phone or email after post-test; muscle strain = patient suffered a rectus femoris muscle strain during exercise and was not able to continue.

Randomization was conducted after clinical assessment of the eligibility criteria, and the first test session. All patients were drawn from 60 sealed opaque envelopes of a 2:1:1 ratio of WLC, HSR, and WBV group. At the end of the initial intervention period, patients of the WLC group drew new assignments to HSR and WBV groups with a 1:1 probability. Randomization procedures were not stratified for potential confounding variables (e.g., symptom duration, sex, site). All envelopes were prepared off-site by FR. All physicians (SH, KL, JH) remained blinded to the patient’s allocation at post-testings and follow-up measurements. Sports scientists (FR, H-PW) were blinded for the baseline assessments (t1) and data analysis but became unblinded for some patients during post-testings. However, an independent encoding of the patient’s identity after enrolment and decoding after finishing data analyses ensured proper blinding also for these situations. The study protocol is shown in detail in Figure 2.

### Intervention protocols

The HSR and WBV interventions consisted of three sessions per week and were followed weekly for 12 weeks by qualified exercise therapists at the Paracelsus Medical University. Heavy slow resistance training sessions consisted of three bilateral knee extension exercises: squats, leg press, and hack squats (barbell and hack squat: Precor, Garching b. München, Germany; leg press: Proxomed, Alzenau, Germany). Each task was performed in 4 sets of 15 repetitions in the first week. Progressive overload was ensured by increasing the weight with a concurrent reduction of repetitions: 12 repetition maximum (RM) in weeks 2 and 3, 10 RM in weeks 4 and 5; 8 RM in weeks 6–8; 6 RM in weeks 9–12 RM. The knee extension was performed over a range of 90° to 0° (with 0° corresponding to full extension) for all exercises and lasted 3 s for the eccentric and three for the concentric phase, as per previously published protocols (Kongsgaard et al., 2009; Kongsgaard et al., 2010). Whole body vibration sessions involved 10 sets of 60 s static standing in a slightly squatted position on an oscillating platform (Galileo, Novotec Medical, Pforzheim, Germany), with 60 s rest between sets. The knee joint angle was standardized at 50° (0° full extension), and vibration parameters were set at a frequency of 30 Hz, amplitude of 2 mm, and acceleration of 2 g. These settings trigger morphological changes in healthy human patellar tendons (Rieder et al., 2016). Perceived pain at the end of each training session was assessed to control that exercises were not increasing symptoms. Pain was allowed to be moderate by reaching 50 points at most on the VAS score and with the condition that it would subside by the following morning (Silbernagel et al., 2007). Patients were allowed to continue to take part in sporting activities during the intervention period if they did not experience more than moderate pain (VAS ≤50 points (Silbernagel et al., 2007)). Patients in the WLC group were encouraged to retain their habitual daily routines and performed the same measurements as the intervention group but did not receive any treatment within this first 12 weeks. Thereafter, waitlist patients were offered to receive one of the interventions on a random basis.

### Outcome measures

Clinical symptoms (main clinical outcome variables), tendon morphology, neovascularisation, isometric knee extension strength and tendon mechanical and material properties (all secondary outcome variables) were measured at baseline and after the 12-week intervention period. Clinical symptoms were additionally assessed 6 months after the intervention in the WBV and HSR groups only (see Figure 2). Patients were asked to refrain from any vigorous activity (e.g., resistance training, running, jumping) for 24 h prior to the testing session and maintaining their regular diet. Outcome measurements in this equivalence trial were similar to previously published protocols (Kongsgaard et al., 2009; Kongsgaard et al., 2010).

### Clinical symptoms

Patients completed a VISA-P questionnaire to assess symptoms and functional limitations (0–100 pts; 100 = no symptoms). This questionnaire is reliable and valid for tendinopathy (Visentini et al., 1998; Lohrer and Nauck, 2011) and was *a priori* defined as main clinical outcome variable. Additionally, maximal perceived pain during the patients’ preferred sports activity was rated on a VAS score (0–10 pts; 10 = maximum pain) before and after the intervention period and 6 months after post-testing (Kongsgaard et al., 2009). Patients were made aware to rate the pain at post- and follow-up tests based on the same activity as at baseline. Both questionnaires were completed without investigator’s assistance.

### Tendon morphology

Patellar tendon length and CSAs were determined from sagittal and axial T1 weighted MR images (3T-Achieva, Philips Healthcare, Eindhoven, Netherlands) using the lower extremity coil and the following parameters: TR/TE 682/20 ms, FOV 100, matrix 528 × 528, slice thickness 3.0 mm, space 3.3 mm. Tendon length was measured from a single midsagittal 3D reconstructed image. The patellar tendon length was defined as the distance between the lower pole of the patella and its tibial insertion. Regional tendon CSAs were assessed at three locations: proximal below the apex of the patella (pCSA), distal at the tibial insertion (dCSA), and at mid-length (mCSA). All MR image reconstructions and postprocessing analyses were performed using RadiAnt DICOM software (RadiAnt DICOM viewer version; 5.0.1, Medixant, Poznan, Poland). Mean patellar tendon CSA was calculated as the average of the CSAs measured at the three locations. The same investigator (YS), blinded to the subject’s identity, analyzed all scans. MRI-based CSA measurements of the patellar tendon have been shown to be reliable in several previous studies (Seynnes et al., 2009; Stenroth et al., 2019).

### Neovascularisation

The frequency of neovascular tendons was recorded by SH using color Doppler. The patellar tendon’s Doppler signal was categorized as neovascular if it presented a vessel in the sagittal plane greater than 1 mm (Cook et al., 2004). As neovascularisation is associated with pain (Cook et al., 2004), this variable was included as a covariate (see statistics).

### Muscle strength, tendon material and mechanical properties

The patients performed the tests of maximal force exertion in the following order: Isometric knee extension, isometric knee flexion, and ramped isometric knee extension tests to determine tendon mechanical and material properties. Strength tests were preceded by a warm-up procedure on a cycling ergometer (Precor 846i, Precor, Woodinville, United States), for 10 min at a submaximal intensity of 1.5 W·kg-1 and a pedal rate of 70 rpm.

### Isometric knee extension strength

The patients were seated on an isokinetic dynamometer (IsoMed 2000 D&R Ferstl GmbH, Hemau, Germany) with a knee angle of 90° and strapped with safety belts, shoulder pads, and straps to avoid motion during testing (Dirnberger et al., 2012). After a few contractions intended for familiarization, maximal strength was measured from two maximal isometric knee extensions with 30 s of rest between trials.

### Tendon mechanical and material properties

Tendon mechanical properties were obtained from separate isometric contractions. After assessing isometric knee extension strength, patients performed two maximal isometric knee flexions, where EMG signals of the biceps femoris and semitendinosus muscles were recorded to estimate co-activation during the subsequent ramp contractions. Standard procedures were followed for skin preparation (Hermens et al., 1999) and surface electrodes positioning (Ag/AgCL; 120 dB, Input impedance: 1,200 GOhm; 10 mm diameter, 22 mm spacing, Biovision, Wehrheim, Germany). Subsequently, isometric ramped contractions with a constant loading rate (50 Nm·s-1) were performed with the knee flexed at 90°, to account for the visco-elastic nature of tendons and improve the reliability of mechanical properties measurements (Kösters et al., 2014). The patients achieved a constant loading rate with visual feedback showing actual and target torque levels. The antagonistic torque produced during ramped knee extension was calculated from the EMG activity of flexor muscles by assuming a linear relationship between EMG activity and torque (Magnusson et al., 2001). The EMG signals were filtered offline using a second-order Butterworth filter with cutoff frequencies of 10 and 300 Hz. The EMG amplitude was calculated as the root mean square of the signal over a 0.5 s period around peak torque and was averaged between the two muscles. Tendon force was calculated by dividing the net extension torque by the tendon moment arm length. Moment arm length was calculated individually from femur length (Visser et al., 1990).

The elongation of the patellar tendon during the isometric ramped contractions was recorded using ultrasonography (LA523, 50 mm array, 10–15 MHz transducer, MyLab25, Esaote, Genoa, Italy). Measurements of tendon elongation were performed offline by tracking the displacement of the patella apex and the tibial antero-superior aspect with software for semi-automatic video analysis (Tracker 4.87, physlets. org/tracker/). Tendon resting length was measured before all contractions as the distance between the apex of the patellar to the tibial insertion while patients sat relaxed without muscular activity with their knees bent at 90°.

Load-deformation data obtained with this procedure were then fitted with a second-order polynomial (all relations retained for analysis had a coefficient of determination (*R* ^2^) higher than 0.96). Tendon stiffness was calculated as the slope of this curve at force levels corresponding to the individual highest 10% of the maximal force, as measured during the ramp contraction trials. Stress was calculated by normalizing tendon force by mean CSA, and strain was obtained as the tendon elongation relative to its resting length. Young’s modulus was calculated as the slope of the stress-strain relationship. The same researchers (FR & HPW), blinded to the subject’s identity, performed and analyzed all strength and tendon mechanical and material properties measurements at the Paris-Lodron University of Salzburg. Test-retest measurements in previous work of our group have shown that these variables can be assessed reliably with a coefficient of variation values of 5.5% for stiffness, 7.1% for Young’s modulus, and 10.0% for strain (Rieder et al., 2016; Wiesinger et al., 2016).

### Statistics

The analysis was based on the intention to treat principle, and, once allocated to a group, all available data were included in the analysis. Missing values were not replaced. Data with non-uniform residuals were analyzed using a log transformation as appropriate. Between-group differences at baseline were tested with a one-way ANOVA. In case of significant main effects, a Tukey corrected post hoc test was performed. Pearson’s Chi^2^ test was used for categorical variables. The effectiveness of WBV and HSR interventions were tested separately against the changes measured in the WLC group. An ANCOVA was conducted on the post-test measurements, with pre-test values as a covariate. Symptom duration (range 4–240 months) (Cook and Purdam, 2009), sex (Knobloch et al., 2010), vascularity (Cook et al., 2004) and whether patients were in the waitlist group or treatment groups, as well as whether they continued or interrupted their athletic training (Visnes et al., 2006; van Ark et al., 2015) were considered as covariates. The effect size partial eta squared (η^2^p) was deemed small when η^2^p > 0.01, medium when η^2^p > 0.06, or large when η^2^p > 0.14.

In a second step, equivalence between the effects of WBV and HSR training was tested by calculating mean differences between the changes (Δ pre-post for all variables and Δ pre-follow-up for main clinical outcomes) induced by the two interventions and their 95% confidence intervals (CI) (Piaggio et al., 2006). In this type of analysis, interventions are deemed equally effective if the CI lies wholly within a set eΔ. On the contrary, equivalence is disproved if the CI lies wholly outside eΔ. When the CI crosses one or both limits of eΔ, results are deemed inconclusive (Piaggio et al., 2006). According to previous literature, the equivalence margins for primary outcomes were set as VISA-P ±13 (Hernandez-Sanchez et al., 2014) and VAS ±1.8 (Todd and Funk, 1996). Since there was no similar reference for our secondary outcomes, we used a conservative approach by calculating the smallest worthwhile change (i.e., 20% of between-subject standard deviation of baseline values (Hopkins et al., 1999)). A Pearson correlation was calculated between changes in VISA-P and VAS scores and changes in tendon properties. Correlation coefficients were rated according to Cohan as weak (*r* = 0.10), moderate (*r* = 0.30) and strong (*r* = 0.50). Statistical analyses were performed using the SPSS software (IBM SPSS Statistics 27; Armonk, United States). Figures were produced using the GraphPad Prism 9.0.0 (GraphPad Software Inc, La Jolla, United States). Unless otherwise stated, results are expressed as mean ± standard deviation (SD). The level of significance was set a *p* = 0.05.

## Results

### Patients

The recruitment process and patient flow is displayed in Figure 2. In total, 52 patients were screened: 11 were excluded because of inclusion or exclusion criteria, and two withdrew their consent. Thirteen patients were randomized to WBV training, 11 to HSR training, and 15 to the WLC group. Following a 12-week intervention period, 14 patients of the WLC group were randomly redistributed to one of the two intervention groups (WBV = 5, HSR = 9). One patient was free of pain after the waiting period, and therefore received no further treatment. Four patients from the HSR group and one patient from the WBV group opted out during the intervention (muscle strain injury during HSR *n* = 1; personal reasons *n* = 4), giving a final sample size of *n* = 17, *n* = 16, and *n* = 15, respectively, for the WBV, HSR and WLC groups. The average training session compliance rate was 81 ± 10% for the HSR group and 84 ± 9% for the WBV group. Patient characteristics are presented in Table 1 and were similar compared with previous published randomized controlled trials (Kongsgaard et al., 2009; Kongsgaard et al., 2010). There were no differences between groups at baseline.

**TABLE 1:** Baseline subject characteristics.

	Heavy-slow resistance		Whole body vibration		Waitlist control		F_(1,45) or_ χ^2^	*p*-value	η^2^p^−^Value
Sex (m/f)	16 (12/4)		17 (15/2)		15 (11/4)		1.326	0.515	
Age (yrs)	29	±6	25	±6	27	±6	1.536	0.226	0.046
Body height (cm)	179	±11	180	±8	178	±8	0.138	0.872	0.006
Body mass (kg)	78	±14	78	±13	78	±15	0.002	0.998	0.000
BMI (kg·m-^2^)	24	±3	24	±3	24	±3	0.061	0.941	0.003
Training experience (years)	8	±6	8	±4	8	±7	0.003	0.997	0.000
Activity level (h/week)	6	±4	5	±5	6	±5	0.102	0.903	0.005
Symptom duration (months)	45	±58	41	±45	45	±48	0.022	0.978	0.001
Location (p/d)	13/3		16/1		13/2		1.262	0.532	
Neovascular (n)	8		9		8		0.042	0.979	
Bilateral (n)	8		13		11		3.035	0.219	

Values are expressed as mean ± SD. BMI, body mass index; p, proximal; d, distal.

### Clinical symptoms

At baseline, VISA-P and VAS did not differ between groups (VISA-P: F_(2,45)_ = 1.01, *p* = 0.373, η^2^p = 0.04; VAS: F_(2,45)_ = 0.61, *p* = 0.547, η^2^p = 0.03). Analysis of covariates of HSR changes against WLC revealed that baseline values (VISA-P: F_(1,26)_ = 11.41, *p* = 0.002, η^2^p = 0.31) and sex (VISA-P: F_(1,26)_ = 6.30, *p* = 0.019, η^2^p = 0.20; VAS: F_(1,26)_ = 4.38, *p* = 0.046, η^2^p = 0.14) significantly affected functional limitation and pain scores. Similarly, in the comparison of WBV against WLC, the effects of the covariate baseline values (VISA-P: F_(1,27)_ = 16.27, *p* < 0.001, η^2^p = 0.38; VAS: F_(1,27)_ = 5.22, *p* = 0.030, η^2^p = 0.16) and sex (VISA-P: F_(1,27)_ = 4.90, *p* = 0.035, η^2^p = 0.15; VAS: F_(1,27)_ = 4.42, *p* = 0.045, η^2^p = 0.14) were significant. To examine the effect of sex in greater detail, we conducted 2 (treatment) × 2 (sex) ANCOVAs, maintaining other covariates. The main effect of sex remained significant (VISA-P, HSR: F_(1,26)_ = 7.17, *p* = 0.011, η^2^p = 0.15, WBV: F_(1,27)_ = 4.99, *p* = 0.034, η^2^p = 0.15; VAS, HSR: F_(1,26)_ = 4.38, *p* = 0.046, η^2^p = 0.14), but there was no interaction effect of sex and treatment (VISA-P, HSR: F_(1,26)_ = 0.19, *p* = 0.831, η^2^p = 0.01; WBV: F_(1,27)_ < 0.01, *p* = 0.961, η^2^p < 0.01; VAS, HSR: F_(1,26)_ = 0.03, *p* = 0.885, η^2^p < 0.01; WBV: F_(1,27)_ = 0.36, *p* = 0.553, η^2^p = 0.06). Adjusted mean VISA-P and VAS scores indicated that women displayed higher functional limitations and pain than men (VISA-P, HSR: 62 (SE = 5) vs. 76 (SE = 3), WBV: 56 (SE = 7) vs. 73 (SE = 3) and VAS, HSR: 5.8 (SE = 0.8) vs. 4.0 (SE = 0.4), WBV: 5.6 (SE = 1.0) vs. 4.2 (SE = 0.4).

**FIGURE 3:**
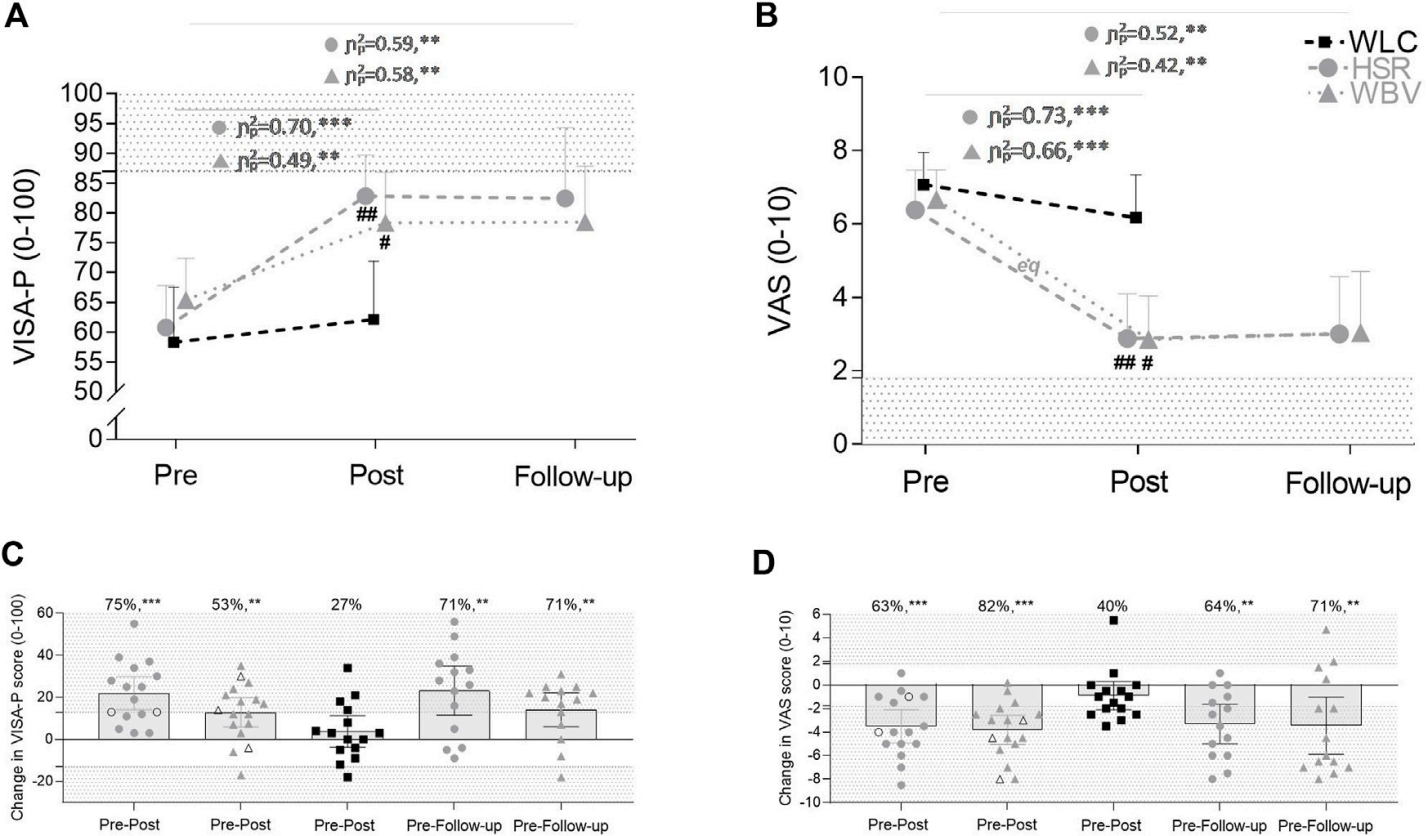
VISA-P and VAS scores at baseline, after 12 weeks of heavy slow resistance (HSR) training, whole body vibration (WBV) or waitlist control (WLC) and 6 months post-treatment phase **(A,B)** and scatter plots of changes in VISA-P scores **(C)** and VAS scores **(D)**. The ranges for minimal clinically relevant effects were set at ± 13 pts for VISA-P (Hernandez-Sanchez et al., 2014) and ±1.8 pts for VAS (Todd and Funk, 1996) scores, with the shaded area representing improvement and deterioration, respectively. In **(C**,**D)**, the individual improvements and the percentage of patients achieving a clinically relevant improvement are demonstrated for the HSR, the WBV and the WLC groups. Empty symbols **(C**,**D)** refer to dropouts after the intervention period. Values are mean ±95% confidence limits. ^#^, *p* < 0.05, ^##^, *p* < 0.01, significantly different to WLC; *eq*, equivalence between WBV and HSR protocols was established from pre-test to post-test. Further, each subgroup was analyzed independently (paired sample *t*-test); **, *p* < 0.01, ***, *p* < 0.001 within groups.

VISA-P values at post-test were significantly greater in HSR (F_(1,26)_ = 10.52, *p* = 0.003, η^2^p = 0.29) and WBV (F_(1,27)_ = 5.94, *p* = 0.022, η^2^p = 0.18) groups compared to WLC, with improvements of +22 ± 15 pts (HSR) and +13 ± 14 pts (WBV). VAS scores were significantly lower after HSR (F_(1,26)_ = 6.50, *p* = 0.005, η^2^p = 0.33) and WBV training (F_(1,27)_ = 3.95, *p* = 0.031, η^2^p = 0.23) training compared to WLC, with decreases of -3.5 ± 2.6 pts and -3.8 ± 2.4 pts, respectively. Improvements in both scores were maintained 6 months after the end of both interventions (Δ pre-follow-up VISA-P: HSR +23 ± 20 pts, WBV +14 ± 14 pts; VAS: HSR -3.3 ± 2.9 pts, WBV -3.5 ± 4.2 pts) (Figure 3).

**FIGURE 4:**
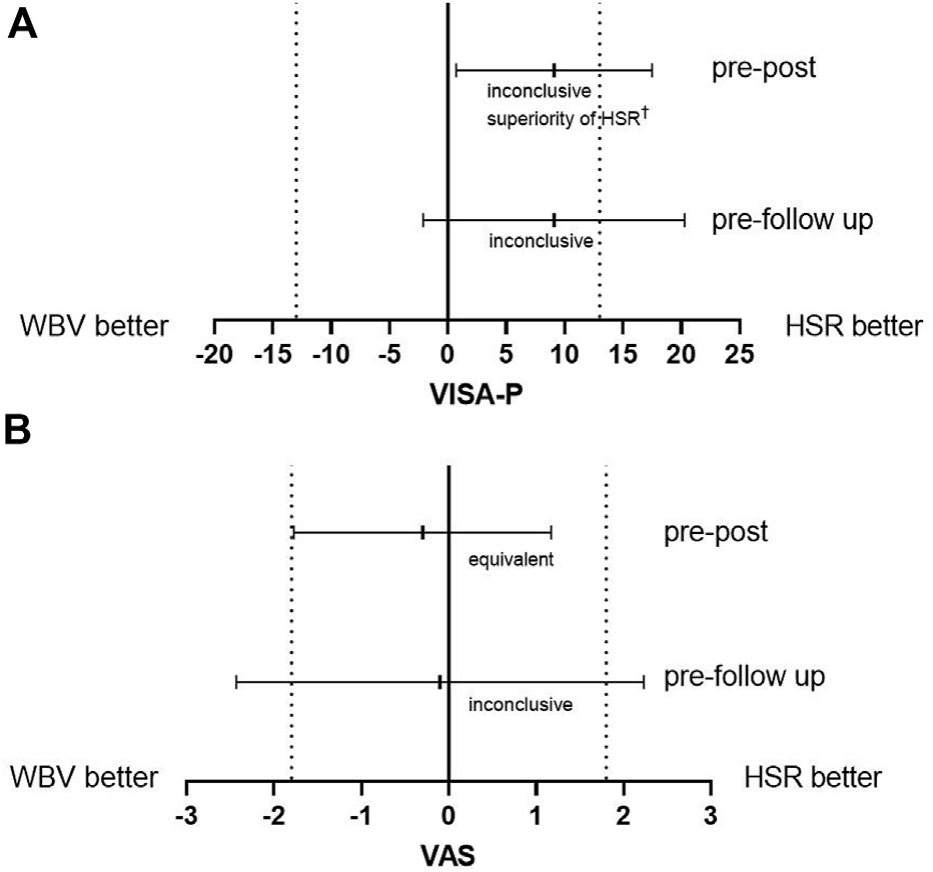
Mean difference of intervention effects from pre-test to post-test and from pre-test to follow-up measurements for **(A)** the VISA-P and **(B)** the VAS scores and the 95% confidence intervals. Dotted lines represent the equivalence margin. HSR = heavy slow resistance training; WBV = whole body vibration training. ^†^ The result is inconclusive regarding possible inferiority of magnitude equivalence margin or worse since the confidence interval includes the equivalence margin (Piaggio et al., 2006).

Pre-post changes of the VAS scores were equivalent between HSR and WBV, and statistically different for the VISA-P score. However, the confidence interval included one delta margin leading to an inconclusiveness for the superiority of HSR group related to the changes in VISA-P (Piaggio et al., 2006) (Figure 4). All other pre-post and pre-follow-up changes were statistically inconclusive (Figure 4).

Training did not affect indicators of tendon degeneration. Only one tendon of the WBV group was rated non-degenerated, with no hypoechoic area, increased signal intensity, or tendon thickening post-treatment. Similar, there were no changes in the number of tendons rated as neovascular, except for one additional neovascular tissue in the HSR group (Pre-test: WBV = 9; HSR = 8; WLC = 8; Post-test: WBV = 9; HSR = 9; WLC = 8).

### Tendon properties

**FIGURE 5:**
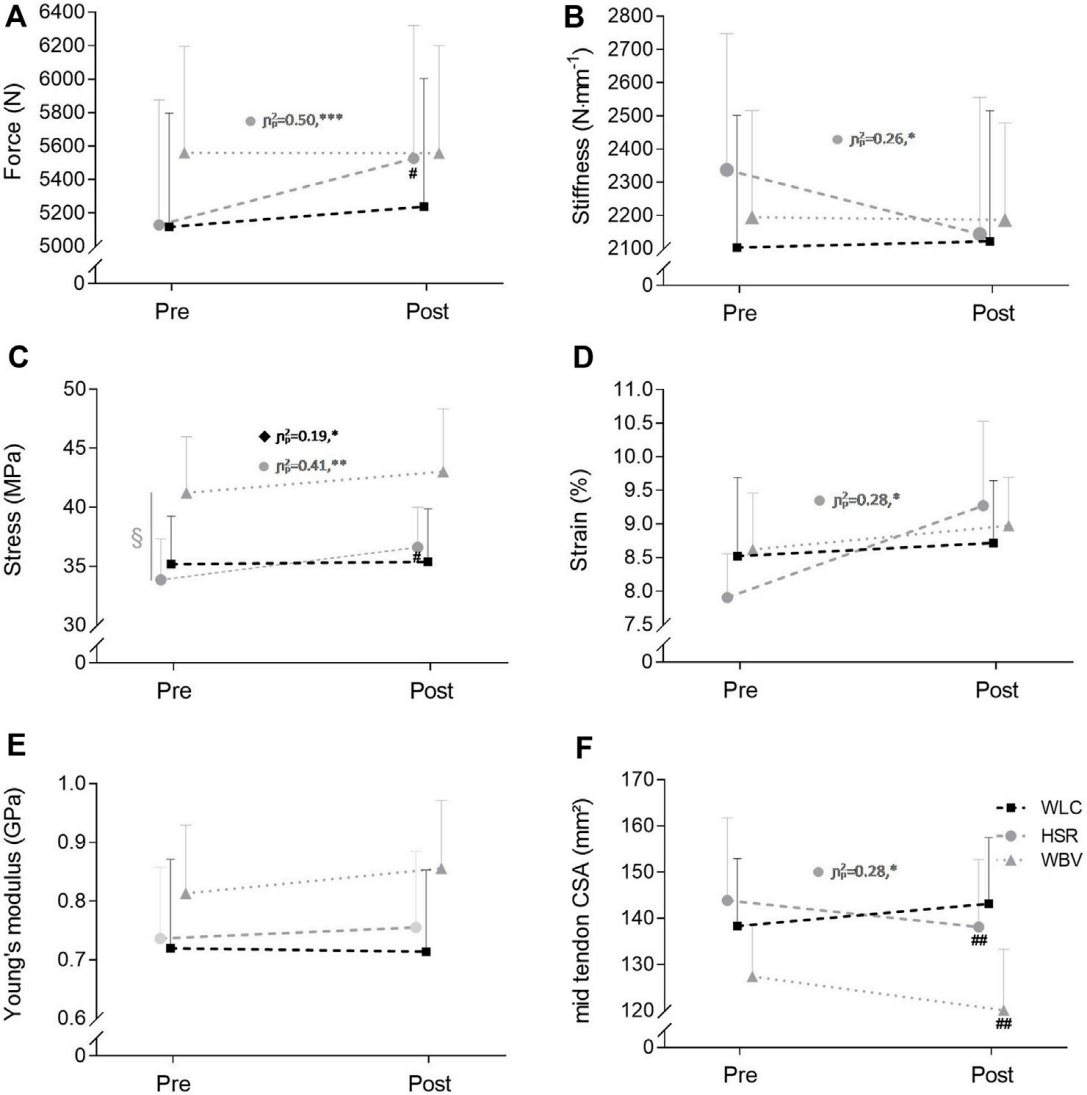
Changes in patellar tendon force **(A)** and patellar tendon properties **(B–F)** of the heavy slow resistance (HSR) training, the whole body vibration (WBV) and the waitlist control (WLC) groups. Values are mean ±95% confidence limits. Statistical analyses of tendon strain were conducted with log-transformed data.; ^§^, *p* < 0.05 significant group effect; ^#^, *p* < 0.05, ^##^, *p* < 0.01, significant different to WLC; CSA = cross-sectional area. Further, each subgroup was analyzed independently (paired sample *t*-test); *, *p* < 0.05, **, *p* < 0.01, ***, *p* < 0.001 within groups.

There were no baseline differences in tendon properties between groups (F_(2,45)_ ≤ 2.04, *p* ≥ 0.142, η^2^p ≤ 0.08), except a significant main effect for stress (F_(2,45)_ ≤ 1.07, *p* = 0.022, η^2^p = 0.16). Stress was 18% higher in the WBV group compared to the HSR group (Q_(2,31)_ = 2.71, *p* = 0.025, η^2^p = 0.18) (Figure 5).

Mid-tendon CSA decreased significantly in both intervention groups, compared to WLC, by -3.0% and -5.7% after HSR and WBV training, respectively. There were no pre-post differences in maximum isometric knee extension strength between HSR (from 193 ± 57 Nm to 210 ± 57Nm) and WLC (from 199 ± 59Nm to 208 ± 65Nm; F_(1,26)_ = 1.39, *p* = 0.249, η^2^ *p* = 0.05) or WBV (from 212 ± 52Nm to 209 ± 26Nm) and WLC (F_(1,27)_ = 2.31, *p* = 0.139, η^2^ *p* = 0.08). Other tendon parameters remained unaffected. The equivalence analysis on the effect of HSR and WBV on tendon properties and maximum isometric knee extension strength remained inconclusive (Table 2 and Figure 5). Tendon tensile force and stress increased significantly after HSR training (+8.5%; +9.1%) compared to WLC (+1.9%; +0.2%). Tendon property values of all groups at baseline and after the intervention period are detailed in a Table in the Supplementary Table S2.

**TABLE 2:** Mean difference of intervention effects with equivalence margins and 95% CI.

	Mean difference	Equivalence margin	95% CI	
MVC torque (Nm)	19.7	±10.9	[5.5; 33.9]	inconclusive^†^
stiffness (N mm^−1^)	−185.2	±138.6	[−396.7; 26.3]	incinclusive
stress (Mpa)	0.9	±1.7	[−1.9; 3.7]	inconclusive
PTF	401.4	±263.7	[138.8; 664.0]	inconclusive^†^
strain (%)	−1.0	±0.3	[−2.1; 0.1]	incinclusive
Young’s modulus (GPa)	0.0	±0.0	[−0.1; 0.1]	inconclusive
mean CSA (mm^2^)	3.8	±5.8	[−2.2; 9.8]	inconclusive
pCSA (mm^2^)	4.9	±8.4	[−3.9; 13.7]	inconclusive
mCSA (mm^2^)	1.6	±5.8	[−6.6; 9.8]	inconclusive
dCSA (mm^2^)	5.1	±6.4	[−2.3; 12.5]	inconclusive
affected CSA (mm^2^)	5.3	±8.7	[−3.4; 14.0]	inconclusive

CI, confidence interval; MVC, maximal voluntary contraction; PTF, patellar tendon force; CSA, cross sectional area; p, proximal; m, mid-tendon; d, distal; affected CSA, CSA, of the affected tendon region.

†statistically significant but the result is inconclusive.

None of the analyses were influenced by choice of covariates, as evidenced by the similar results obtained with and without their inclusion (data not shown).

There was evidence of a statistically significant moderate positive association between changes in maximum voluntary contraction torque and VISA-P (r = 0.305, *p* = 0.035). There was a tendency of an association between changes in patellar tendon stiffness and VISA-P (r = -0.282, *p* = 0.052). There was no evidence of other statistically significant associations between changes in tendon parameters and clinical symptoms. All results of the association analyses are reported in the relevant Table in the Supplementary Table S3.

## Discussion

This clinical trial investigated the use of WBV in the management of chronic patellar tendinopathy. With a particularly rigorous design, long-lasting clinical benefits of both WBV and HSR training were shown beyond the wait-and-see effects. Comparing both interventions, WBV is equally effective as HSR training in reducing maximal pain. Vibration also leads to clinically relevant improvements in functional limitation (Δ VISA-P > 13 pts), but HSR might be superior. Ultrasonographic features of tendon abnormality did–as expected–not change, with the notable exception of mid-tendon CSA in both treatment groups.

### Clinical symptoms

The baseline severity of patellar tendinopathy and the effects of both interventions on clinical symptoms are in line with previous intervention studies using strength training regimes (Kongsgaard et al., 2009; Kongsgaard et al., 2010; Lee et al., 2020). Both WBV and HSR interventions improved the VAS and the VISA-P scores compared to WLC, although only pre-post changes in VAS were equivalent between both treatment groups. The likely different effect between HSR and WBV on functional limitations cannot be elucidated from the present data. We ascribe this to differences in contraction type and loading intensity. Typical loading regimes in the current or previous HSR interventions (Kongsgaard et al., 2009; Kongsgaard et al., 2010; Lee et al., 2020) involve heavy resistance over a broad range of motion, while patients in the WBV group experienced a quasi-isometric muscle contraction. This hypothesis fits with recent findings showing no clinically meaningful improvements in functional limitations after 4 weeks of isometric exercises in patellar tendinopathy patients (van Ark et al., 2015). Accordingly, the patellar tendon may experience less loading and strain during vibration exercises. Follow-up tests demonstrated the durability of improvements in functional limitations after 6 months for 71% of patients in either intervention group. Similarly, a clinically relevant reduction in maximum perceived pain post-intervention was evident in 71% of the WBV group and 64% of the HSR group after 6 months. Of note, this is the first study comparing loading-based treatments to a WLC group in patients suffering from patellar tendinopathy. Including a control condition is important to avoid an overestimation of observed treatment effects. Time-dependent reliefs or seasonal changes in activity patterns (e.g. reduced training load in the off-season or different impact loads by changing from indoor volleyball to beach volleyball) can also influence pain perception. Confirming this risk of bias, many of our WLC patients achieved clinically relevant improvements in the VISA-P (27%) and VAS (40%) scores. On the other hand, a minority of WLC patients (7%) experienced a worsening of clinical symptoms. Even though improvements in mean VISA-P and VAS scores did not reach significance for this group, including an inactive control group in the research design limited the chances of type I error and overestimating effect sizes (see Figure 4).

### Tendinopathy features and neovascularization

The number of tendons rated as tendinopathic did not change substantially after the intervention period. The discrepancy between reduced clinical symptoms and unchanged signs of tendinopathy (e.g., swelling and hypoechoic areas) is consistent with previous work (Docking and Cook, 2018). In that respect, the effects of WBV did not produce a different outcome than HSR. In line with Cook et al. (2004), about half of the pathological patellar tendons were rated as presenting neovascularity. As tissue vascularity has been associated with more significant functional limitations (Cook et al., 2004), this variable was entered as a covariate. However, neovascularization was not significantly related to treatment effects, and further investigations tailored to answer this hypothesis are needed.

### Tendon morphology

Exercise interventions affected tendon morphology. After treatment, we observed a reduction in the mid-section CSA for WBV and HSR groups. To the best of our knowledge, only a few studies quantified changes in the CSA of affected patellar tendons after treatment, indicating no significant difference (Kongsgaard et al., 2010; Agergaard et al., 2021) or an increase in the mid-tendon area (Kongsgaard et al., 2009). Tendon enlargement is frequent in patients with tendinopathy and is attributed to an altered extracellular matrix composition (Kongsgaard et al., 2009), including an increased resting water content within the tendinopathic tendon (Ho and Kulig, 2016). Although a reduction in tendon CSA is interpreted as an improvement in this context, additional studies, including tissue sampling, are needed to clarify the links between CSA changes and the healing process.

### Tendon mechanical and material properties and muscle strength

None of the treatment interventions affected tendon mechanical and material properties. The only exception was an increased tendon stress after HSR training, which was a simple mathematical effect of the increased force in this group. Calculations of tendon force were obtained from ramped contractions, which may not necessarily yield maximal force exertion in all subjects. For this reason, the maximal knee extension torque measured in a separate test–without ramped pattern–probably reflects force production capacity more accurately. However, there were no improvements for this parameter in neither of the two training groups compared to the WLC condition. An unchanged maximum strength after WBV fitted the results obtained in our previous study in healthy subjects and was anticipated (Rieder et al., 2016). The reason why isometric strength did not change for the HSR group either is unclear. Most studies reporting the effect of heavy slow resistance training typically included a ramped isometric test (e.g. Kongsgaard et al., 2009; Kongsgaard et al., 2010; Agergaard et al., 2021) or conducted dynamic strength tests (e.g., Ruffino et al., 2021). The present results suggest a poor transfer between dynamic strengths gains and this isometric test. These results markedly differ from the nearly systematic stiffening of non-symptomatic tendons after resistance training (Wiesinger et al., 2015). In this sense, they may fit with a characteristic spectrum of responses to exercise training tending to have no effect or even decrease tendon stiffness (Kongsgaard et al., 2009; Kongsgaard et al., 2010; Lee et al., 2020). Few studies (Kongsgaard et al., 2009; Kongsgaard et al., 2010; Lee et al., 2020) have examined the effect of therapies on patellar tendon stiffness in tendinopathy, and the interpretation of what may be seen as maladaptation is unclear. It is hypothesized that tendon healing and associated remodeling, perhaps including a temporary reorganization of cross-links, may cause a reduction in stiffness (Kongsgaard et al., 2010). Associations between reductions in tendon stiffness and improvements in the VISA-P score, observed in this study for the combined cohort of treated patients and earlier (Lee et al., 2020), tend to support this hypothesis. Interdisciplinary studies using direct measures of tendon remodeling are needed to clarify the evolution of tendon mechanical properties in developing tendinopathy and recovering from it.

## Limitations

Before concluding, possible limitations should be considered. Although we recorded and statistically controlled whether patients continued recreational or athletic activities, we cannot rule out that exercise volume, duration, or intensity changes have affected the clinical outcomes. Furthermore, we lack experimental approaches to determine the smallest worthwhile changes in various tendon properties on function, pain or performance outcomes. The chosen threshold of 0.2 SDs of all subjects in the pre-test is objective in the sense that it is defined by the data. However, the clinical relevance of changes in tendon properties is yet to be established, and the relation between the reduction in pain and tissue healing is unknown. Additional research is required to define clinical relevance thresholds.

## Conclusion

Whole body vibration seems equally effective as HSR in reducing maximum pain in patients suffering from chronic patellar tendinopathy. It also leads to long-lasting functional improvements in most patients. The present results suggest that HSR training should remain the first choice in treating patellar tendinopathy. However, WBV could be recommended in patients in whom the latter is contraindicated. In addition, vibration may be used at the onset of treatment to reduce maximal pain sufficiently for the patients to start HSR training more effectively. Based on the results of this study and the anabolic effect of vibration on healthy human tendons (Rieder et al., 2016), the preventive potential of these exercises should be further investigated.

## Data Availability

The raw data supporting the conclusion of this article will be made available by the authors, without undue reservation.
