# Supplementary material for: Whole body vibration for chronic patellar tendinopathy: A randomized equivalence trial
Source: Front Physiol. 2022 Oct 21;13:1017931. doi: 10.3389/fphys.2022.1017931 (PMC9633993; doi:10.3389/fphys.2022.1017931)
Supplement: Supplementary file 1 [file Table1.docx]

Supplementary Material

# Supplementary Tables

Table 1: Consort Checklist

CONSORT Statement 2006 - Checklist for Non-inferiority and Equivalence Trials
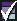


**Items to include when reporting a non-inferiority or equivalence randomized trial**

| ***PAPER SECTION* And topic** | 1. **Item** | **Descriptor** | **Reported on**  **Page #** |
| --- | --- | --- | --- |
| 1. *TITLE & ABSTRACT* | 1 | [How participants were allocated to interventions](http://www.consort-statement.org/index.aspx?o=1107) (*e.g*., "random allocation", "randomized", or "randomly assigned"),  s*pecifying that the trial is a non-inferiority or equivalence trial.* | 2 |
| *INTRODUCTION* Background | 2 | [Scientific background and explanation of rationale](http://www.consort-statement.org/index.aspx?o=1016),  *including the rationale for using a non-inferiority or equivalence design.* | 2 |
| *METHODS* Participants | 3 | [Eligibility criteria for participants](http://www.consort-statement.org/index.aspx?o=1017#3a) *(detailing whether participants in the non-inferiority or equivalence trial are similar to those in any trial(s) that established efficacy of the reference treatment)* and the [settings and locations where the data were collected](http://www.consort-statement.org/index.aspx?o=1017#3b). | 3&7 |
| Interventions | 4 | [Precise details of the interventions intended for each group *detailing whether the reference treatment in the non-inferiority or equivalence trial is identical (or very similar) to that in any trial(s) that established efficacy,* and how and when they were actually administered](http://www.consort-statement.org/index.aspx?o=1021). | 5 |
| Objectives | 5 | [Specific objectives and hypotheses](http://www.consort-statement.org/index.aspx?o=1022), *including the hypothesis concerning non-inferiority or equivalence*. | 2 |
| Outcomes | 6 | [Clearly defined primary and secondary outcome measures](http://www.consort-statement.org/index.aspx?o=1023#6a) *detailing whether the outcomes in the non-inferiority or equivalence trial are identical (or very similar) to those in any trial(s) that established efficacy of the reference treatment* and, when applicable, any [methods used to enhance the quality of measurements](http://www.consort-statement.org/index.aspx?o=1023#6b) (*e.g.*, multiple observations, training of assessors). | 5ff |
| Sample size | 7 | [How sample size was determined](http://www.consort-statement.org/index.aspx?o=1024#7a) *detailing whether it was calculated using a non-inferiority or equivalence criterion and specifying the margin of equivalence with the rationale for its choice*. When applicable, [explanation of any interim analyses and stopping rules](http://www.consort-statement.org/index.aspx?o=1024#7b) (*and whether related to a non-inferiority or equivalence hypothesis*). | 3 |
| Randomization -- Sequence generation | 8 | [Method used to generate the random allocation sequence, including details of any restrictions](http://www.consort-statement.org/index.aspx?o=1025) (*e.g*., blocking, stratification) | 4f |
| Randomization -- Allocation concealment | 9 | [Method used to implement the random allocation sequence](http://www.consort-statement.org/index.aspx?o=1026) (*e.g*., numbered containers or central telephone), clarifying whether the sequence was concealed until interventions were assigned. | 4f |
| Randomization -- Implementation | 10 | [Who generated the allocation sequence, who enrolled participants, and who assigned participants to their groups](http://www.consort-statement.org/index.aspx?o=1027). | 4f |
| Blinding (masking) | 11 | [Whether or not participants, those administering the interventions, and those assessing the outcomes were blinded to group assignment](http://www.consort-statement.org/index.aspx?o=1028#11a). If done, [how the success of blinding was evaluated](http://www.consort-statement.org/index.aspx?o=1028#11b). | 4f |
| Statistical methods | 12 | [Statistical methods used to compare groups for primary outcome(s)](http://www.consort-statement.org/index.aspx?o=1029#12a), *specifying whether a one or two-sided confidence interval approach was used*. [Methods for additional analyses](http://www.consort-statement.org/index.aspx?o=1029#12b), such as subgroup analyses and adjusted analyses. | 6f |
| 1. *RESULTS*   Participant flow | 13 | [Flow of participants through each stage](http://www.consort-statement.org/index.aspx?o=1018) (a diagram is strongly recommended). Specifically, for each group report the numbers of participants randomly assigned, receiving intended treatment, completing the study protocol, and analyzed for the primary outcome. [Describe protocol deviations from study as planned, together with reasons](http://www.consort-statement.org/index.aspx?o=1086). | Figure 2 |
| Recruitment | 14 | [Dates defining the periods of recruitment and follow-up](http://www.consort-statement.org/index.aspx?o=1087). | 2 |
| Baseline data | 15 | [Baseline demographic and clinical characteristics of each group](http://www.consort-statement.org/index.aspx?o=1088). | 7 and Table 1 |
| Numbers analyzed | 16 | [Number of participants (denominator) in each group included in each analysis and whether the analysis was](http://www.consort-statement.org/index.aspx?o=1089) *“intention-to-treat”* *and/or* *alternative analyses were conducted*. State the results in absolute numbers when feasible (*e.g*., 10/20, not 50%). | Figure 2, page 6and7 |
| Outcomes and estimation | 17 | [For each primary and secondary outcome, a summary of results for each group, and the estimated effect size and its precision](http://www.consort-statement.org/index.aspx?o=1090) (*e.g.*, 95% confidence interval). *For the outcome(s) for which non-inferiority or equivalence is hypothesized, a figure showing confidence intervals and margins of equivalence may be useful*. | 7ff, Figure 3-5 and Table 2 and Supplemental table 2 and 3 |
| Ancillary analyses | 18 | [Address multiplicity by reporting any other analyses performed](http://www.consort-statement.org/index.aspx?o=1091), including subgroup analyses and adjusted analyses, indicating those pre-specified and those exploratory. | 7ff |
| Adverse events | 19 | [All important adverse events or side effects in each intervention group](http://www.consort-statement.org/index.aspx?o=1092). | 7 and Table 2 |
| *DISCUSSION* Interpretation | 20 | [Interpretation of the results](http://www.consort-statement.org/index.aspx?o=1019), taking into account the *non-inferiority or equivalence hypothesis and any other* study hypotheses, sources of potential bias or imprecision and the dangers associated with multiplicity of analyses and outcomes. | 9ff |
| Generalizability | 21 | [Generalizability (external validity) of the trial findings](http://www.consort-statement.org/index.aspx?o=1094). | 12 |
| Overall evidence | 22 | [General interpretation of the results in the context of current evidence](http://www.consort-statement.org/index.aspx?o=1095). | 9ff |

[**www.consort-statement.org**](http://www.consort-statement.org)

Table 2: Tendon properties before (PRE) and after (POST) the intervention period in the whole body vibration (WBV), heavy slow resistance (HSR) and waitlist control (WLC) groups.

|  | HSR | | | | | | |  | WBV | | | | | | |  | WLC | | | | | | |  | HSR vs WLC | | | WBV vs WLC | | | |
| --- | --- | --- | --- | --- | --- | --- | --- | --- | --- | --- | --- | --- | --- | --- | --- | --- | --- | --- | --- | --- | --- | --- | --- | --- | --- | --- | --- | --- | --- | --- | --- |
|  | PRE | | | POST | | | Δ  [95% CI] |  | PRE | | | POST | | | Δ  [95% CI] |  | PRE | | | POST | | | Δ  [95% CI] |  | F_(1,26)_ | P-Value | *η^2^_p_-Value* | F_(1,27)_ | P-Value | *η^2^_p_-Value* |  |
| MVC torque (Nm) | 193 | ± | 57 | 210 | ± | 57 | 17  [6, 27] |  | 212 | ± | 52 | 209 | ± | 26 | -3  [-16, 9] |  | 199 | ± | 59 | 208 | ± | 65 | 8  [1, 16] |  | 1.39 | .249 | .05 | 2.31 | .139 | .08 |  |
| PTF (N) | 5128 | ± | 1404 | 5526 | ± | 1493 | 398  [198, 598] |  | 5560 | ± | 1237 | 5556 | ± | 1254 | -3  [-230, 223] |  | 5115 | ± | 1229 | 5237 | ± | 1385 | 122  [-19, 263] |  | 4.70 | .039 | .15 | 0.76 | .391 | .03 |  |
| Strain (%) | 7.9 | ± | 1.2 | 9.3 | ± | 2.4 | 1.4  [0.3, 2.5] |  | 8.6 | ± | 1.6 | 9.0 | ± | 1.4 | 0.4  [-0.4, 1.1] |  | 8.5 | ± | 2.1 | 8.7 | ± | 1.7 | 0.2  [-0.6, 1.0] |  | 1.13 | .297 | .04 | 0.87 | .360 | .03 |  |
| Stress (MPa) | 34 | ± | 7 | 37 | ± | 6 | 3  [1, 4] |  | 41 | ± | 9 | 43 | ± | 10 | 2  [-1, 5] |  | 35 | ± | 7 | 35 | ± | 8 | 0  [-1, 1] |  | 5.23 | .030 | .16 | 1.23 | .276 | .04 |  |
| Stiffness (N·mm^-1^) | 2337 | ± | 771 | 2144 | ± | 774 | -193  [-359, -28] |  | 2194 | ± | 626 | 2186 | ± | 568 | -8  [-187, 170] |  | 2103 | ± | 718 | 2122 | ± | 711 | 18  [-150, 187] |  | 2.21 | .149 | .08 | 0.13 | .720 | .01 |  |
| Modulus (GPa) | 0.7 | ± | 0.2 | 0.6 | ± | 0.2 | 0.0  [0.0, 0.1] |  | 0.8 | ± | 0.2 | 0.9 | ± | 0.2 | 0.0  [0.0, 0.1] |  | 0.7 | ± | 0.3 | 0.7 | ± | 0.3 | 0.0  [-0.1, 0.0] |  | 0.47 | .500 | .02 | 1.31 | .261 | .05 |  |
| Proximal tendon CSA (mm²) | 166 | ± | 41 | 168 | ± | 43 | 3  [-4, 10] |  | 162 | ± | 45 | 160 | ± | 45 | -2  [-9, 6] |  | 164 | ± | 41 | 167 | ± | 41 | 3  [-3, 9] |  | 0.17 | .897 | <.01 | 0.93 | .342 | .03 |  |
| Mid-tendon CSA (mm²) | 144 | ± | 34 | 138 | ± | 27 | -6  [-10, -1] |  | 127 | ± | 22 | 120 | ± | 26 | -7  [-15. 1] |  | 138 | ± | 26 | 143 | ± | 26 | 5  [0, 10] |  | 8.95 | .006 | .26 | 10.27 | .004 | .27 |  |
| Distal tendon CSA (mm²) | 145 | ± | 34 | 146 | ± | 36 | 1  [-5, 7] |  | 123 | ± | 27 | 118 | ± | 30 | -5  [-11, 2] |  | 140 | ± | 39 | 139 | ± | 33 | -1  [-6, 3] |  | 0.56 | .462 | .02 | 1.19 | .285 | .04 |  |
| Affected tendon CSA (mm²) | 168 | ± | 42 | 172 | ± | 46 | 4  [-2, 10] |  | 166 | ± | 46 | 165 | ± | 47 | -1  [-9, 7] |  | 170 | ± | 44 | 171 | ± | 42 | 1  [-5, 8] |  | 0.48 | .494 | .02 | 0.29 | .595 | .01 |  |
| Mean CSA (mm²) | 152 | ± | 32 | 151 | ± | 31 | -1  [-4, 3] |  | 137 | ± | 25 | 133 | ± | 27 | -5  [-10, 1] |  | 147 | ± | 29 | 150 | ± | 28 | 2  [-1, 6] |  | 0.87 | .360 | .03 | 4.51 | .043 | .14 |  |

Values are expressed as means ± SD. PTF: patellar tendon force; MVC: maximal voluntary contraction; CSA: cross-sectional area; affected tendon CSA: CSA of the affected tendon region.

Table 3: Associations between changes in clinical symptoms and changes in tendon properties.

|  | PTF (N) | Strain (%) | Stress (MPa) | Stiffness (N⋅mm^-1^) | Modulus (GPa) | MVC torque (Nm) | pCSA (mm²) | mCSA (mm²) | dCSA (mm²) | meanCSA (mm²) | affectedCSA (mm²) |
| --- | --- | --- | --- | --- | --- | --- | --- | --- | --- | --- | --- |
| VAS  (0-10) | -.021 (P=.888)  95% CI  [-.31, .26] | -.193 (P=.189)  95% CI  [-.47, .08] | .025 (P=.865)  95% CI  [-.26, .31] | .139 (P=.347)  95% CI  [-.14, .42] | .141 (P=.340)  95% CI  [-.14, .42] | -.012 (P=.934)  95% CI  [-.30, .27] | -.077 (P=.604)  95% CI  [-.36, .21] | .084 (P=.571)  95% CI  [-.20, .37] | -.109  (P=. 461)  95% CI  [-.39, .17] | -.042 (P=.779)  95% CI  [-.33, .24] | -.086 (P=.560)  95% CI  [-.37, .20] |
| VISA-P  (0-100) | .267 (P=.067)  95% CI  [.01, .54] | .189 (P=.199)  95% CI  [-.08, .47] | .211 (P=.150)  95% CI  [-.06, .49] | **-.282 (P=.052)**  **95% CI**  **[-.55, -.03]** | -.204 (P=.164)  95% CI  [-.48, .07] | **.305 (P=.035)**  **95% CI**  **[.06, .57]** | .124 (P=.402)  95% CI  [-.16, .41] | -.171 (P=.246)  95% CI  [-.45, .10] | .074 (P=.615)  95% CI  [-.21, .36] | .009 (P=.950)  95% CI  [-.28, .29] | .191 (p=.194)  95% CI  [-.08, .47] |

CI: confidence interval; PTF: patellar tendon force; MVC: maximum voluntary contraction; pCSA: proximal cross sectional area; mCSA: mid-tendon cross sectional area; dCSA: distal cross sectional area; affected CSA: CSA of the affected tendon region.
